# Cerebral Venous Thrombosis in a Young Male Patient With HIV AIDS

**DOI:** 10.7759/cureus.76665

**Published:** 2024-12-31

**Authors:** Shrushti J Tanna, Anandu M Anto, Victoria Lovallo, Eghosa Omoregie, Misbahuddin Khaja

**Affiliations:** 1 Internal Medicine, BronxCare Health System, Bronx, USA; 2 Medicine, BronxCare Health System, Bronx, USA; 3 Internal Medicine, Ross University School of Medicine, Bridgetown, BRB; 4 Neurology, BronxCare Health System, Bronx, USA

**Keywords:** cerebral venous and dural sinus thrombosis, dural venous sinus, hiv aids, left vein of trolard, seizure

## Abstract

Dural venous sinus thrombosis (DVST) is characterized by thrombosis in the cerebral veins, the dural venous sinus, or both. Headache is the most common initial symptom. Patients can present with seizures, papilledema, focal neurological deficits, and mental status alteration. We describe a case of a young HIV-positive patient with a seizure disorder who presented with unrelenting headaches and breakthrough seizures and was diagnosed with thrombosis in the superior sagittal sinus extending to the left transverse and sigmoid sinuses with venous thrombosis in the left vein of Trolard. This case is highly relevant, reminding clinicians to keep a high index of suspicion for DVST in any patient with headache and neurological manifestations.

## Introduction

Dural venous sinus thrombosis (DVST) is the formation of a blood clot in the cerebral sinuses. It can occur in all age groups but is common in young to middle-aged adults [1]. The clot forming in the dural venous sinus occludes the outflow through the sinuses, resulting in backpressure and elevated venous pressure and may lead to infarction or hemorrhage [2]. Depending on the severity, symptoms can be a spectrum ranging from mild headache to severe headache and altered mental status with neurological deficits such as limb weakness, vision changes, or changes in speech. We present the case of a 31-year-old HIV-positive male patient who had an episode of seizure and was diagnosed with DVST in the superior sagittal sinus, the left transverse and sigmoid sinuses, and the left vein of Trolard. 

## Case presentation

A 31-year-old male patient with a medical history of seizure disorder not adherent to treatment, cirrhosis secondary to alcohol use disorder, and HIV/AIDS (not adherent to combined antiretrovirals) was brought to the emergency department by Emergency Medical Services (EMS) after being found unconscious on the floor of his home. The patient experienced a seizure that lasted approximately 10 seconds, as witnessed by EMS personnel.

**Figure 1:**
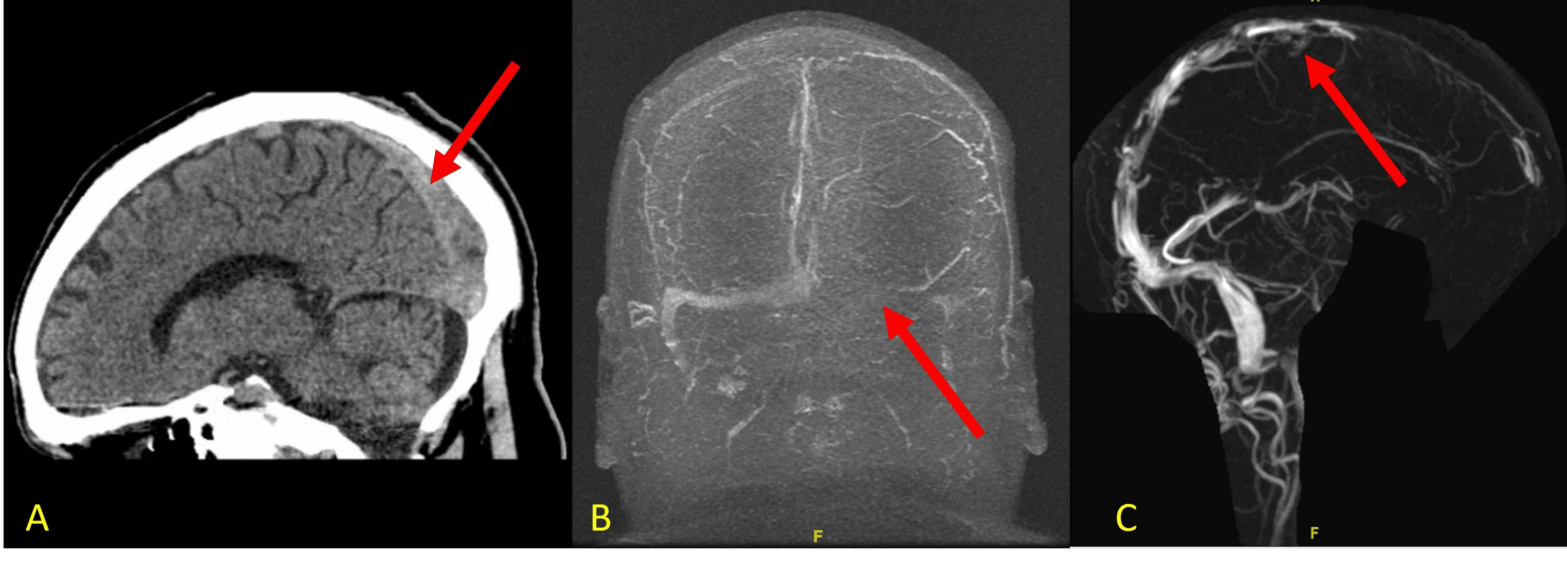
Imaging CT: computed tomography; MR: magnetic resonance (A) CT head showing decreased density within the superior sagittal. (B and C) MR venogram showing dural venous thrombosis in the superior sagittal sinus, extending to the left transverse, sigmoid sinuses, and left vein of Trolard

Upon arrival at the emergency department, the patient's mental status improved, but he complained of a severe headache. A computed tomography (CT) scan of the head showed no acute hemorrhage or cerebrovascular accident (CVA) but demonstrated decreased density within the superior sagittal and bilateral transverse sinuses, raising suspicion for venous sinus thrombosis (Figure 1). Vital signs were within normal limits. On examination, the patient was alert, oriented to time, place, and person, and exhibited no neurological deficits.

Laboratory tests upon admission revealed mild transaminitis and an elevated ethanol level of 268 mg/dL (Table 1). The patient was initiated on a heparin drip as per neurology's recommendations for suspected DVST, received chlordiazepoxide for alcohol withdrawal, and was started on antiepileptic therapy. He was subsequently admitted to the hospital.

**Table 1:** Laboratory data RBC count: red blood cell count; WBC count: white blood cell count; BUN: blood urea nitrogen; ALP: alkaline phosphatase; AST: aspartate aminotransferase; ALT: alanine aminotransferase; APTT: activated partial thromboplastin time; PT: prothrombin time; CD4 count: cluster of differentiation 4 count; CD8: cluster of differentiation 8

Lab test	Result	Reference range
RBC count	4.66	4.50-5.90 MIL/ul
WBC count	4.9	4.8-10.8 k/ul
Hemoglobin	14.7	12.0-16.0 g/di
Hematocrit	44	42.0-51.0%
Platelet	337	150-400 k/ul
Sodium	145	135-145 mEq/L
Potassium	4.1	3.5-5.0 mEq/L
Chloride	104	98-108 mEq/L
BUN	4	8.0-26.0 mg/dL
Creatinine	0.7	0.5-1.5 mg/dL
Total bilirubin	1.0	0.2-1.2 mg/dL
Direct bilirubin	0.2	0.0-0.3 mg/dL
ALP	186	53-128 unit/L
AST	119	9-48 unit/L
ALT	97	5-40 unit/L
PT	11	10.4-15.7 second
INR	0.91	0.85-1.29
APTT	30.2	28.2-42.9 second
Serum ethanol level	268	<= 10.0 mg/dL
Serum levetiracetam level	<2.0	6.0-46.0 mcg/mL
Serum methylmalonic acid	100	55-335 nmol/L
Homocysteine total	8	<11.4 umol/i
Protein C functional assay	81	70-180% normal
Protein S functional assay	93	70-150% normal
Antithrombin III assay	85	80-135% normal
Antiphospholipid Ab panel	Negative	
Factor V Leiden mutation analysis	Variant not detected	
Prothrombin 20210A mutation	Negative	
BCR ABL 1 gene QN PCR	Not detected	
Cardiolipin Ab screen	Negative	
Myeloproliferative neoplasm core DX panel	No mutations were detected in codon 617 or exon 12 of JAK2, or exon 9 of CALR, or exon 10 (codons 505 and 515) of MPL	
HIV 1/2 O Ab	Reactive	
HIV RNA ON PCR copies	9250 copies/mL	Target not detected
CD4%	24	30-61%
Absolute CD4 cells	171	490-1740 cells/uL
CD4/CD8 T cell ratio	0.48	0.86-5.00

Magnetic resonance venography (MRV) and MR imaging (MRI) of the head and neck confirmed the diagnosis of dural venous thrombosis in the superior sagittal sinus, extending to the left transverse and sigmoid sinuses. Venous thrombosis was also observed in the left vein of Trolard (Figure 1). Hematology recommended transitioning from heparin to apixaban. Hypercoagulable workup was negative (Table 1). After a detailed discussion of the risks and benefits, apixaban was started, considering the patient’s history of alcohol dependence and recurrent falls.

The patient's symptoms improved after three days of therapy and Librium tapered off. He was discharged to an alcohol rehabilitation program and educated about medication adherence. Follow-up appointments were arranged.

## Discussion

DVST is a relatively rare finding, contributing to only 0.5-2% of stroke cases [3]. The prognosis is often good, but it is still a grave condition that can lead to serious neurological deficits as well as coma and death if not identified and promptly treated [4]. The clinical manifestations of DVST are widely varied, making it difficult for clinicians to suspect it. The most common manifestations seen are severe headaches (90%), seizures (47%), hemiparesis (43%), papilledema (41%), impaired consciousness (39%), and coma (15%) [4].

The heightened and ongoing state of inflammation caused by HIV contributes to the development of thromboses [5,6]. HIV-positive patients are around two to ten times more likely to develop venous thrombosis [7,8]. Unregulated HIV causes significant immune stimulation and leads to ongoing high levels of inflammatory cytokines [5]. HIV infection permanently damages intestinal lymphatics, leading to increased translocation of microbial products from the gut, which, in turn, contributes to monocyte activation [9]. Monocyte activation results in the upregulation of tissue factors, severe endothelial dysfunction, cytokine-induced liver dysfunction, and reduced nitric oxide production [6,10,11]. A small case-control trial to determine the relevance of protein S deficiency in HIV patients by Mochan et al. in 2005 found a statistically significant association between protein S deficiency and stroke, concluding that protein S deficiency in HIV-positive patients with stroke is an epiphenomenon of the HIV infection [12]. Similarly, protein C has also been found to be low in HIV patients making them at high risk for thromboembolism [13].

The key to understanding the pathophysiology in our case lies in the findings of Baker et al. [6]. They proposed that HIV-infected patients who are not receiving combined antiretroviral therapy exhibit abnormalities in coagulation factors, which seem to be dependent on the function of hepatocytes. Our patient was a chronic alcoholic, which caused persistent hepatocyte injury. Declining hepatic function in the setting of HIV (nonadherent to therapy) resulted in a procoagulant state, leading to a dural venous sinus thrombus. This procoagulant state due to HIV persists until the end stage of the liver disease, at which point it leads to an almost complete lack of coagulation factors, resulting in hemorrhagic manifestations [6]. The association of HIV and prothrombotic state is so strong that some researchers assert that positive HIV status is sufficient to consider prophylaxis and long-term treatment for venous thromboembolism regardless of low risk in the context of young age and absent inherited risk factors [8]. Treatment of HIV with antiretroviral therapy improves coagulation physiology by reducing inflammation and improving antithrombin III production by the liver [6].

Chronic alcohol use is known to alter other mechanisms of coagulation. Chronic alcoholism results in frank and/or functional B12 deficiency [14]. Chronic alcohol use by itself or in conjunction with B12 deficiency can result in hyperhomocysteinemia, leading to increased thrombotic risk [15,16]. Heavy alcohol use is associated with impaired fibrinolysis and enhanced platelet activation [17]. A retrospective cohort study observed that people who have been hospitalized with alcohol intoxication are significantly more likely to develop thromboses on follow-up compared to people who have not been hospitalized for alcohol-related reasons [18].

Laboratory imaging aids in clarifying the diagnosis of DVST. The neuroimaging techniques used are CT/CT venography, MRI/MRV, and cerebral angiography [6]. MRI/MRV is the gold standard for diagnosing DVST because it can show the thrombus and demonstrate the degree of reduction in blood flow. Treatment involves anticoagulation (heparin/warfarin/direct oral anticoagulants (DOACs)) to prevent thrombus progression and potentially recanalize the sinus. The ACTION-CVT trial [19] evaluated the use of DOACs versus warfarin for cerebral venous thrombosis and found that the clinic-radiological outcomes and safety profile were similar. Patients with antiphospholipid antibody syndrome will be treated with warfarin after two randomized trials demonstrated an increase in arterial thrombotic events in patients treated with rivaroxaban compared with warfarin [20]. Catheter-based thrombectomy with or without fibrinolysis may be considered for patients who do not respond to anticoagulation, as studies have shown that patients did not benefit from endovascular treatment when compared with standard anticoagulation [21].

Our case is challenging because of the preexisting seizure disorder and active alcohol abuse, which may lead to a heuristic and anchoring bias labeling this as a seizure due to alcohol and nonadherence to antiseizure medications. Therefore, this serves as a gentle yet powerful reminder to consider all possible differential diagnoses for seizures in HIV and AIDS patients, including dural venous sinus thrombosis, which is a rare pathology in this population.

## Conclusions

DVST is a rare potential cause of headaches, seizures, and strokes but can have devastating outcomes if not identified and treated in a timely manner. There are challenges to identifying DVST, such as the variability in clinical manifestation and the subtle radiographic findings. However, keeping a high degree of suspicion of DVST, especially in the context of HIV, can aid in timely identification and intervention, reducing morbidity and mortality.
